# The growth performance of pond-reared common carp (*Cyprinus carpio*) larvae propagated using cryopreserved sperm

**DOI:** 10.1007/s10695-023-01245-x

**Published:** 2023-10-03

**Authors:** Zoltán Bokor, Zete Levente Láng, Levente Várkonyi, Ferenc Fodor, Borbála Nagy, Endre Csókás, József Molnár, Balázs Csorbai, Zsolt Csenki-Bakos, Bence Ivánovics, Jeffrey Daniel Griffitts, Béla Urbányi, Gergely Bernáth

**Affiliations:** 1 Department of Aquaculture, Institute for Aquaculture and Environmental Safety, Hungarian University of Agriculture and Life Sciences, 2100 Godollo, Hungary; 2 Balaton Fish Management Non-Profit Ltd, Horgony U. 1., 8600 Siofok, Hungary; 3 Department of Environmental Toxicology, Institute for Aquaculture and Environmental Safety, Hungarian University of Agriculture and Life Sciences, 2100 Godollo, Hungary

**Keywords:** Common carp, Cryopreserved sperm, Propagation, Pre-nursing and grow-out, Survival rate

## Abstract

The aim of our study was to determine the efficacy of utilizing cryopreserved common carp sperm (in comparison to fresh sperm) for propagation at a Hungarian aquaculture facility. The sperm was frozen in 5 mL straws using an extender method that was previously tested in common carp. Sperm motility was monitored using a computer-assisted sperm analysis system. The hatching and malformation rates among the specimens were recorded before the stocking of larvae in both groups. The growth (body weight, total length) and survival rates of the fish were measured during the pre-nursing (from May to June: between 1 and 26 days post hatching) and grow-out periods (from June to October: between 26 and 105 days post hatching) of the same year. The fresh sperm, which was collected and pooled prior to fertilization, showed high MOT (97%), pMOT (92%), VCL (106 µm s^−1^), LIN (75%), and ALH (1.84 µm). Prior to the fertilization trial of the cryopreserved sperm, low MOT (34%), pMOT (14%), and VCL (61 µm s^−1^) values were observed in frozen-thawed sperm. A significantly higher hatching rate was measured in the fresh sperm group (87%) when compared to the cryopreserved sperm group (42%). No significant difference in the overall malformation rate was observed in larvae originating from either the fresh or frozen sperm. A significant difference between the two test groups was observed in the incidence of deformed tails (fresh: 20%, cryopreserved: 55%). Except for one sampling period, no significant difference in the body weight and total length of the fish larvae was found between the two groups throughout the pre-nursing and grow-out periods. A significantly higher larvae survival rate was noted in the fresh sperm (72%) as compared to the cryopreserved group (43%) by the end of the pre-nursing stage. However, no significant difference in survival rate was observed for the cryopreserved sperm (96%) in comparison to the fresh sperm (95%) by the end of the grow-out stage. The results of this study showed, for the first time in large-scale pond culturing, an equal growth and viability in larvae propagated from cryopreserved sperm when compared to fresh sperm (despite the limited available rearing ponds provided by the commercial company).

## Introduction

The aquaculture industry produces fish and other aquatic products that provide nearly 20% of the protein intake for one-third of the world’s human population. The common carp (*Cyprinus carpio*) is the third most farmed fish in the world, and in central and eastern Europe, it is the most cultivated fish (almost exclusively within ponds) (Adámek et al. 2019). Cyprinid fish are traditionally cultured in extensive and semi-intensive polyculture systems (Adámek et al. 2019; Guillen and Motova 2013). The research and development of carp cultivation methods that improve the vital aspects of controlled reproduction (e.g. reproductive physiology, hormonal stimulation, artificial propagation, sperm and egg production quality) began around 70 years ago (Chattopadhyay 2016; Marinović et al. 2021). Large numbers of advanced fry can be profitably produced in ponds where the appropriate quantity and quality of a natural food source is available for the stocked larvae and developing fry (Horváth et al 2015). In natural conditions, the survival rate of the fish is relatively low and is heavily dependent on the availability of food and the presence or absence of predators. Comparatively, in cultured conditions, fish survival can be enhanced with the use of some appropriate management strategies (physical and biological preparation of ponds, stocking of feeding larvae, monitoring of fish growth and health, harvesting process, etc.) (Horváth et al. 2015). These procedures have been largely standardized over the past four decades, resulting in the feeding conditions are being nearly identical between different hatcheries, although some variability still exists. There are inherent problems within aquaculture systems, such as inbreeding, genetic drift, and introgressive hybridization, all of which negatively influence fish production (Betsy et al. 2021). To counter some of these issues associated with aquaculture, the utilization of spermbanks can support the conservation of genetically pure common carp lines and enhance their controlled reproduction (Marinović et al. 2021; Martínez-Páramo et al. 2017).

Effective sperm cryopreservation methods have been developed in recent decades due to the increasing number of potential applications in (1) aquaculture (genetic improvement programs, broodstock management, propagation in species with reproductive problems, asynchrony in gamete production), (2) biotechnology studies utilizing model fish species (preservation of transgenic or mutant lines), (3) the cryobanking of genetic resources (endangered species, species with high ecological and angling value) (Asturiano et al. 2017; Bernáth et al. 2018; Cabrita et al. 2010; Horváth et al. 2012). Due to its economic and ecological value, the common carp is one of the most intensively studied freshwater species for the implementation of long-term preservation methods (Bernáth et al. 2016; Moczarski 1977). Several laboratories have achieved high post-thaw motility and high fertilization rates on a small scale at experimental conditions (Bernáth et al. 2016; Fauvel et al. 2010; Várkonyi et al. 2019). Despite the optimization of freezing protocols, thawed carp sperm is still not routinely used in industrial hatcheries during the production process. A number of papers have emphasized the need for the development of cryopreservation methods which can be implemented in industrial practices and are able to accommodate increased volumes of sperm and large-scale fertilization (Bernáth et al. 2016; Horváth et al. 2007; Lahnsteiner et al 2003; Sotnikov et al. 2023; Várkonyi et al. 2019). A crucial need in the development of a carp cryopreservation protocol is research into the health and physiology of hatched larvae originating from cryopreserved sperm (Horváth et al. 2007). According to our knowledge, the growth and survival rate of carp larvae obtained from cryopreserved sperm has been not investigated in traditional extensive pond cultures.

The first aim of this study was to implement cryopreserved common carp sperm in large-scale hatchery propagation (fertilization of 1 kg of eggs). The second aim of this study was to compare the growth and survival rate of larvae obtained from both fresh and frozen sperm during the pre-nursing and grow-out periods in a traditional, extensive pond culture at a commercial Hungarian fish farm.

## Materials and methods

### Broodstock management

For sperm cryopreservation, a male broodstock (*N* = 13, standard length: 44 ± 3 cm, body weight: 1875 ± 371 g, purchased from the Balatoni Halgazdálkodási Nonprofit Ltd. fish farm in Buzsák, Hungary) was maintained in a recirculating aquaculture system (RAS) within the Department of Aquaculture at the Institute for Aquaculture and Environmental Safety, Hungarian University of Agriculture and Life Sciences. The system contains a 1-m^3^ tank with water change rate 1500 L per hour. The used water flowed through the bottom outlet by gravity onto a 500 × 500 mm 5-cm-thick TM 35 filter sponge (density: 22 kg/m^3^, cell size 1600–2200 μm). From there it flowed to the biofilter with a net volume of 0.125 m^3^, in which 60 L of biomedia with a total surface area of 800 m^2^/m^3^ and a protected surface area of 500 m^2^/m^3^ provided for the bacteria carrying out the biofiltration. The air flow through the biofilter was 750 L per hour. The system also included a 40-W UV lamp and a 0.5-m^3^ equalization tank. Fish were kept in 1 m^3^ polypropylene tanks (temperature: 19 °C, dissolved oxygen: 8.4 mg L^−1^, stable balanced photophase duration (light:dark 12:12)). The individuals were fed with 100 g per day dry feed (Aqua Garant UNI 4 mm, Aqua Garant GmbH, Austria) body weight. The broodstock for fertilization experiments was maintained at the Balatoni Halgazdálkodási Nonprofit Ltd. fish farm. Males (*N* = 6, body weight: 8100 ± 1980 g) and females (*N* = 9, body weight: 9100 ± 3300 g) were kept in concrete tanks in a flow-through system at approximately 20 ± 3 °C (varied during the rearing period according to the input water source).

### The hormonal stimulation and collection of gametes

Fish were anaesthetized with 2-phenoxyethanol (99%, Sigma-Aldrich, St. Louis, USA) at a dose of 0.4 ml L^−1^ (Bernáth et al. 2018). Based on our former study, males (*N* = 13, for sperm cryopreservation) kept in a RAS were injected using dissolved (0.9% NaCl solution) carp pituitary (purchased from a fish farm) at a single dose of 2.5 mg body weight kg^−1^ (Várkonyi et al. 2019). The water temperature was raised to 20 °C following injection. The individuals maintained at the hatchery of the fish farm were hormonally stimulated (followed the maintainer’s hatchery practice) using 1 pellet body weight kg^−1^ of OVOPEL ((D-Ala^6^, Pro^9^NEt)-mGnRH + metoclopramide), Interfish Ltd., Budapest, Hungary). Males (*N* = 6, for fresh sperm controls) were injected with 1 dose of OVOPEL 24 h prior to gamete collection. Females were stimulated with two doses of OVOPEL: 1, 10% 24 h, 2, 90% 12 h before the expected ovulation. Males were stripped (24 h post hormonal stimulation) using a 5-mL syringe and sperm was stored at 4 °C before the experimental procedures of this study. Eggs were collected in plastic bowls and gametes were stored at hatchery temperature (25 °C). Sperm and eggs were collected avoiding contamination with mucus, faeces and water. Gametes were stored for approximately 30 min before application.

### Sperm motility assessment

The total motility (MOT, %), progressive motility (criteria according to the Sperm VisionTM v. 3.7.4.- threshold: straight line distance > 5 µm s^−1^, pixel to µm ratio: 151 to 100, pMOT %), curvilinear velocity (VCL, µm s^−1^), linearity (LIN, %), and amplitude lateral head displacement (ALH, µm) of fresh and thawed sperm were measured using a CASA (Computer-assisted Sperm Analysis) system (Sperm VisionTM v. 3.7.4., Minitube of America, Venture Court Verona, USA) (Várkonyi et al. 2019). Spermatozoa were activated (ratio: 1:50) at a temperature 20 °C with a simple saline solution (45 mM NaCl, 5 mM KCl, 30 mM Tris, pH: 8.0 ± 0.02, Saad et al. 1988) designed for common carp in a mixture with 0.01 g mL^−1^ bovine serum albumin (BSA, 98%, Sigma-Aldrich, St. Louis, USA) on Makler counting chamber (depth: 10 µm, Sefi Medical Instruments Ltd., Haifa, Israel). Measurements were carried out (in 15 s) at minimum in duplicates, where moving cells were captured (cell identification range: 1 to 100 µm^2^) with a digital camera (JAI CV-A10 CL, Minitube of America, Venture Court Verona, USA) using a frame rate of 60 s^−1^.

### Cryopreservation method

The cryopreservation of common carp sperm was carried out earlier prior day of the propagation (to reduce storage time of egg) in the laboratory of the Department of Aquaculture at the Hungarian University of Agriculture and Life Sciences. Following the motility analysis of the male broodstock (*N* = 13), 9 males (showing the highest pMOT values in a range of 73–97%) were selected for cryopreservation. The total volume of samples (*x̄* = 9 mL per sample, *N* = 9) were pooled and diluted using a modified sugar and ion based extender (205 mM glucose, 20 mM NaCl, 25 mM KCl, 1 mM Na_2_HPO_4_ • 12H_2_O, 1 mM MgCl_2_ • 6H_2_O, 1 mM CaCl_2_ • 2H_2_O, 20 mM Tris and 0.5% BSA, pH: 8.0 ± 0.02; Molnár et al. 2020a, b; Várkonyi et al. 2019), previously designed for pike and common carp by our group, at a ratio of 1:9 (sperm:extender + cryoprotectant). As a cryoprotectant, a 10% methanol solution was used. Diluted sperm were loaded into 5 mL straws (4 mL total volume of sample) and frozen in a Styrofoam box (internal dimensions of height: 22 cm, width: 25.5 cm, length: 36 cm, and the thickness of the walls 2.5 cm) for 7 min at 3 cm above the level of liquid nitrogen (Bokor et al. 2010; Várkonyi et al. 2019). Altogether, 103 straws (~ 41 mL of fresh sperm) were preserved for fertilization of an elevated amount of eggs. Frozen sperm was stored in a 10 L transport canister tank (Statebourne Cryogenics, United Kingdom). All chemicals were purchased from Reanal (Budapest, Hungary) and Sigma-Aldrich (Budapest, Hungary). Straws were thawed under hatchery conditions for 35 s at 40 °C using a bucket with aquarium heaters (Bokor et al. 2010; Várkonyi et al 2019). Straws were used for fertilization immediately following thawing.

### Propagation process

**Fig. 1:**
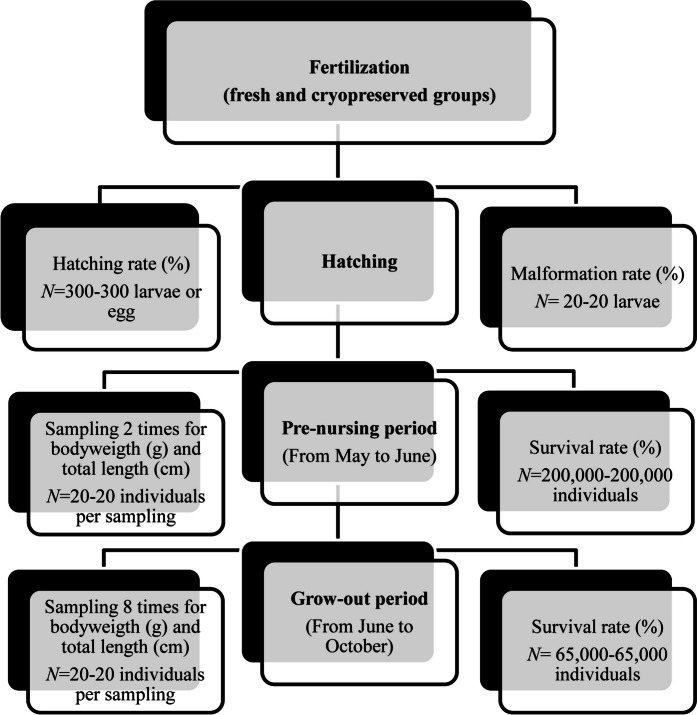
The graphical abstract of the experimental design

The experiment was incorporated to the seasonal propagation period of the fish farm. Fish propagation was carried out following the maintainer’s protocol at the Balatoni Halgazdálkodási Nonprofit Ltd. hatchery in Buzsák. As a first step, 4.5 kg of eggs were collected and pooled from 4 females. Fresh sperm was stripped from 5 males and pooled. Motility was recorded according to the abovementioned method (2.3.). Egg batches (1 kg for fresh and 1 kg for cryopreserved group) were fertilized using both fresh (25 mL) and thawed sperm (250 mL: containing 25 mL sperm, 225 mL extender + methanol). Sperm were activated using hatchery water (100 mL, 20 °C, 198 mosmol kg^−1^). A traditional Woynárovich solution (68 mM NaCl and 50 Mm urea, Woynárovich and Woynárovich 1980) was used to remove the stickiness of the egg. The eggs were incubated (using Woynárovich solution) for 90 min using a horizontal shaker (HS 501 Digital Plus Horizontal Shaker, IKA, Germany) at 60 rpm. The final step of the procedure was the application of the tannin solution (0.5 g L^−1^) in triplicate (1: 20 s, 2:15 s, 3:10 s). Between each step, the batches were washed with pure hatchery water (Woynárovich and Woynárovich 1980). The maintainer provided two separate 100 L Zuger jars (according to the availability). The fresh and cryopreserved groups were incubated separately at 20 °C for 3.5 days up to hatching. The hatching rate (hatched larvae per total egg number) was visually calculated using bright field stereo microscope at the moment of hatching (Fig. 1; 300 ~ 300 individuals or eggs, Bokor et al. 2019). During sampling, a reduced number of individuals were collected based on the request of the hatchery manager (no influencing production process).

### Larval morphology

The evaluation of larval malformation (curved body, deformed tail development, yolk-sac deformity, head deformity, oedema, aberrant swim bladder, aneurysm haemorrhage) was carried out as described in our previous study (Bernáth et al. 2018). During sampling, a reduced number of individuals were collected based on the request of the hatchery manager (no influencing production process). At hatching, 20 randomly selected (based on our former study) individuals from both the fresh and cryopreserved groups were examined (Molnár et al. 2020a, b). The larvae were anaesthetized with tricaine methane sulfonate (MS222, 168 mg L^−1^, Matthews and Varga 2012). The body of the fish was fixed using a methylcellulose solution (3%). Pictures were taken using a Leica DFC 7000 T digital camera connected to a Leica M205 FA stereomicroscope using a bright field setting. During this process, a 20-fold magnification and 80 ms shutter speed were used. Fish were visually investigated using Leica Application Suite X software (Leica Microsystems GmbH; Wetzlar, Germany). All chemicals were purchased from Reanal (Budapest, Hungary) and Sigma-Aldrich (Budapest, Hungary). Malformation rate was calculated in overall and type of the malformation separately (%, malformed larvae per 20 individuals; Fig. 1).

### Larval rearing

The experiment was incorporated to the seasonal rearing period of the fish farm. The larval rearing was separated for two periods, following the maintainer’s protocol. The maintainer provided ponds (according to the availability) for the pre-nursing (2 ponds) and grow-out (2 ponds) period separately. The pre-nursing was carried out for 3 weeks (from May to June: between 1 and 26 days post hatching) in 2 (0.1 ha) earthen ponds. From both fresh and cryopreserved groups, 200,000 (400,000 total) individuals were stocked in two different ponds (Pond fresh: water temperature 22.5 ± 1.3 °C, dissolved oxygen 16.2 ± 0.64 mg L^−1^; pond cryo:water temperature 22.3 ± 1.6 °C, dissolved oxygen 16.5 ± 0.72 mg L^−1^). Larvae were fed using Hungarian commercial dry feed (Ponty előnevelő táp II., Haltáp Ltd., Szarvas, Hungary). Grow-out was performed in two 0.5 ha earthen ponds (from June to October: between 26 and 105 days post hatching). In this period, 65,000–65,000 fingerlings were stocked in two different ponds (pond fresh: water temperature 17.2 ± 5.5 °C, dissolved oxygen 11.1 ± 0.82 mg L^−1^; pond cryo:water temperature 17.6 ± 5.2 °C, dissolved oxygen 11.3 ± 0.56 mg L^−1^). The individuals utilized the natural food supply (zooplankton, balanced availability during the experiment) and were also fed using commercial dry feed (Aller Aqua master 2 mm, Aller Aqua, Denmark). The natural food supply was monitored by the colleagues of the fish farm.

During both rearing periods, regular growth parameters (20 individuals per group, Anton-Pardo et al. 2014; Hartvich et al. 2003; USAID 2011) were measured at different intervals (pre-nursing: at the stocking and harvesting (2 times), grow-out: in two weeks interval (8 times) (Fig. 1). A reduced number of individuals were collected based on the request of the hatchery manager (no influencing production process). The body weight (g) was measured with two types of scale (Mettler Toledo AB204-S, Mettler Toledo, USA and KERN EMB 200–3, KERN & SOHN GmbH, Germany) according to the size of the fish. Total length (cm) was measured using a ruler. The survival rate (%, all surviving individual per stocking number) was recorded at harvesting, following the pre-nursing and grows-out (2 times). All fish was harvested using traditional fishing net where caught individuals were counted using a 200-mL beaker (Fig. 1).

### Statistical analysis

The data were analysed using the SPSS 22.0 (SPSS Inc., Chicago, USA) and GraphPad Prism 8.01 for Windows (GraphPad Software, La Jolla, California, USA) software. The normal distribution was evaluated with a Kolmogorov–Smirnov test at a significance level of *P* < 0.05. The dataset not showing normal distribution was transformed using a logarithmic (total length and body weight) function. Different groups of total length and average body weight were compared using two-way ANOVA followed by Bonferroni’s post hoc test (significance level: *P* < 0.05). Hatching, larvae malformation, and survival rate were compared with an odds ratio test (significance level: *P* < 0.05).

## Results

Pooled fresh sperm samples, prior to cryopreservation, exhibited high MOT (88%), pMOT (79%), VCL (102 µm s^−1^), LIN (77%), and ALH (1.63 µm). In the fertilization trial, a low MOT (34%), pMOT (14%), and VCL (61 µm s^−1^) values of frozen sperm were observed following thawing. In LIN (80%) and ALH (1.67 µm) parameters, the reduction was not apparent. Fresh sperm, collected and pooled prior to fertilization, showed high MOT (97%), pMOT (92%), VCL (106 µm s^−1^), LIN (75%), and ALH (1.84 µm).

**Fig. 2:**
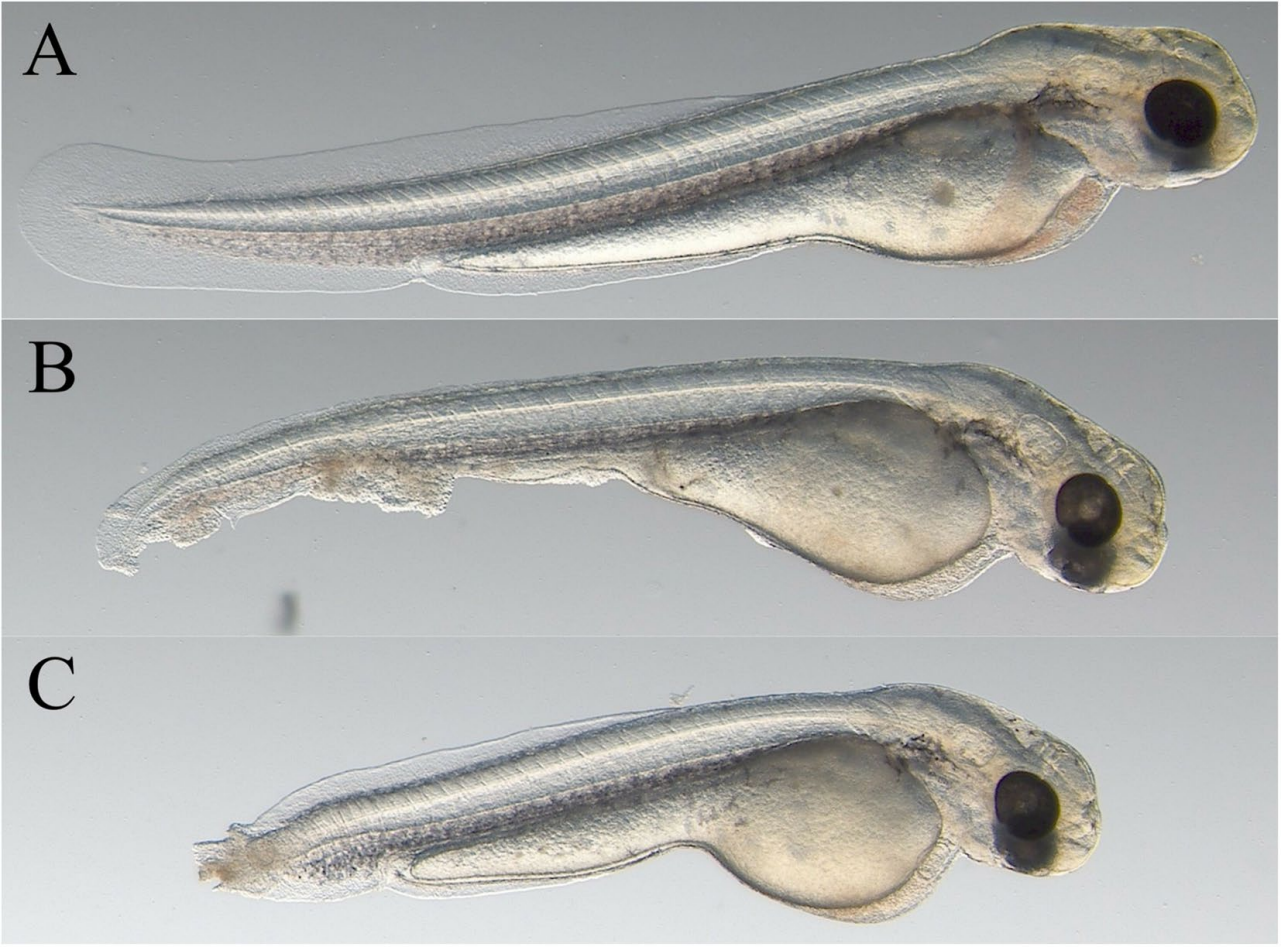
Representative developmental dysfunctions of carp larvae obtained from fresh and cryopreserved groups following hatching. **A** Normal development, **B**–**C** deformed tail development and head deformity (*N* = 20)

A significantly higher hatching rate was measured in the case of the fresh group of sperm (87%) compared to the cryopreserved sperm (42%) (odds ratio: 10.3455, *P* < 0.0001). No significant difference (odds ratio: 2.2698, *P* = 0.2069) in overall malformation rate was recorded in both fresh and frozen groups (Table 1). Larvae malformations occurred in many cases jointly by the investigated individuals. The prevalence of deformed tail development (fresh: 20%, cryopreserved: 55%) and head deformity (fresh: 35%, cryopreserved: 50%) was the most conspicuous malformation in both the control and cryopreserved groups (Fig. 2A–C). Significant difference was observed only in the deformed tail development (odds ratio: 4.8889, *P* = 0.0269). Head deformity did not show significant difference between fresh and cryopreserved groups (odds ratio: 1.8571, *P* = 0.3394).

**Table 1:** The hatching (*N* =  ~ 300–300 larvae or egg), malformation (*N* = 20–20 larvae), and survival rate (*N* pre-nusing = 200,000–200,000, *N* grow-out = 65,000–65,000) in fresh and cryopreserved groups. Larvae malformation rate was analysed at the moment of hatching. The table represents mean values. The different letters correspond to a significant difference between the fresh and cryopreserved groups at the given parameters

	Fresh	Cryopreserved
Hatching rate (%)	87**a**	42**b**
Larvae malformation rate (%)	45**a**	65**a**
Survival rate (pre-nursing, %)	72**a**	43**b**
Survival rate (grow-out, %)	95**a**	96**a**

**Fig. 3:**
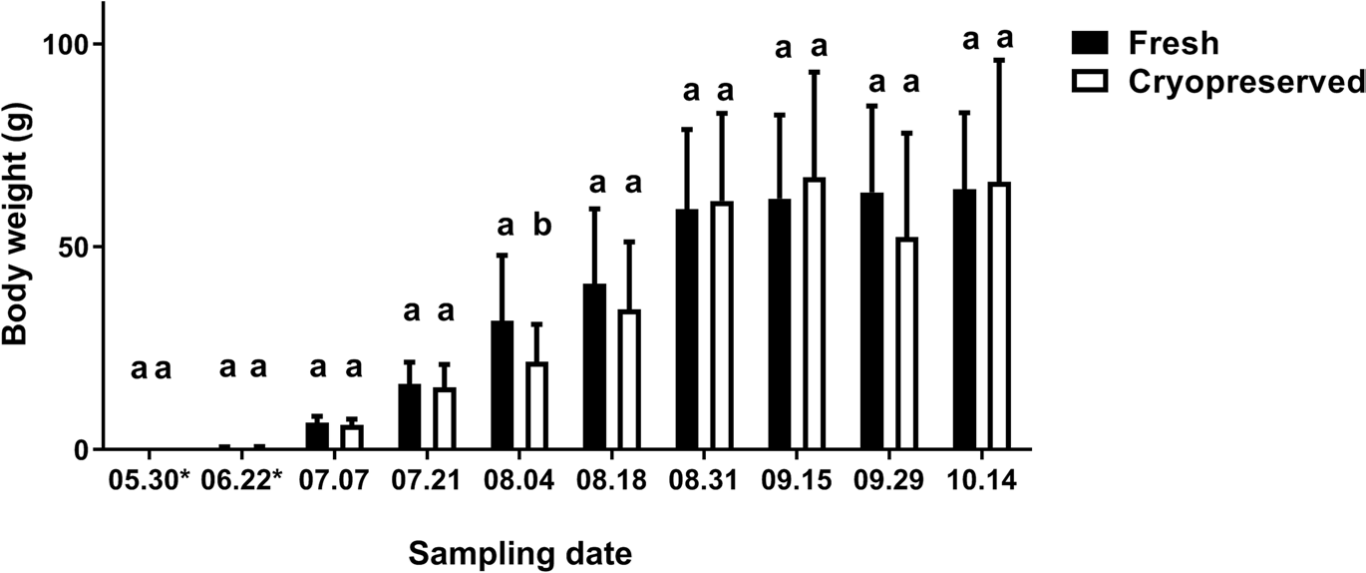
The body weight (g) measured in fresh and cryopreserved groups (*N* = 20–20 per sampling date) during the pre-nursing (marked with asterisk) and grow-out periods. The columns represent the mean and standard deviation. Different letters represent a significant difference between the two groups at each given sampling date

**Fig. 4:**
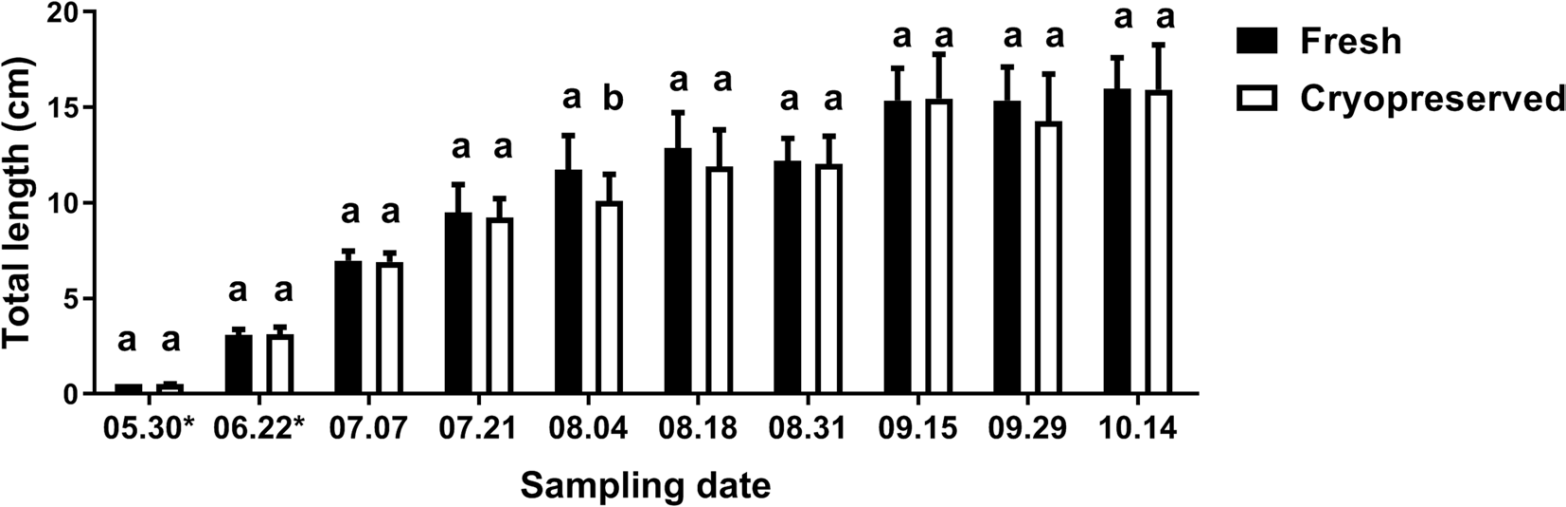
The total length (cm) measured in the fresh and cryopreserved groups (*N* = 20–20 per sampling date) during the pre-nursing (marked with asterisk) and grow-out periods. The columns represent the mean and standard deviation. Different letters represent a significant difference between the two groups at each given sampling date

In both fresh and cryopreserved groups, obvious developmental progression in body weight (*P* = 0.0103) and total length (*P* = 0.0021) of larvae was noted at the 31st of August and the 15th of September time points. Except for the sampling on the 4th of August (body weight: *P* < 0.05, total length: *P* < 0.01), no significant difference was measured in the body weight and total length of larvae between the two groups (Figs. 3 and 4). Significantly higher larvae survival rate was found in the fresh (72%) compared to the cryopreserved group (43%) at the end of pre-nursing (odds ratio: 3.4790, *P* < 0.0001). Contrary, no significant difference in survival rate was calculated for the cryopreserved sperm (96%) in comparison to the fresh sperm (95%) at the end of the grow-out stage (odds ratio: 0.8155, *P* = 0.245) (Table 1).

## Discussion

Despite of the unique farm environment and the special requirements of the fish farm during the production process (lowered jar and pond repetition, reduced sample size in the experiments), novel tendencies in hatching, malformation, and growth rate were recorded following application of cryopreserved sperm in traditional pond culture.

In the present study, the application of our formerly developed (Várkonyi et al. 2019), large-scale cryopreservation method was successfully implemented in hatchery conditions for the fertilization of an industrial volume of eggs (1 kg). To assess the effectiveness of our cryopreservation method, the growth and survival rates of larvae were monitored throughout the pre-nursing and grow-out periods. A clear negative effect of cryopreservation and thawing on spermatozoa movement was observed. This result is in contrast with our former study when the method was optimized for common carp. Várkonyi et al. (2019) used a similar extender and Styrofoam box method for 5 mL straws, resulting in a ~ fourfold higher pMOT (64 ± 8%) with similar VCL (64 ± 14 µm s^−1^) in comparison with our study (pMOT: 14%, VCL: 61 µm s^−1^). According to our hypothesis, the formerly developed thawing method (Várkonyi et al. 2019) was optimal for a few individual 5 mL straws; however, the thawing of 100 straws, as in our study, at the same time caused irreversible damage to the frozen cells (Denniston et al. 2011; Piironen 1993; Tiersch 2011). Further industry innovations are needed in order to develop specialized equipment which can handle such a high volume of large straws while maintaining a stable environment during the thawing process. Furthermore, Várkonyi et al. (2018) proved that carp sperm could be cryopreserved at a large-scale (10 mL cryotube) using a dilution ratio of 1:1. A lower dilution ratio with larger freezing capacity can reduce the adequate straw number. Furthermore, low dilution rate facilitates a sufficient high sperm concentration for fertilization of large egg volumes (Sotnikov et al. 2023).

In comparison with former studies, the decrement in MOT, pMOT, and VCL could affect hatching rate drastically in our case. Horváth et al. (2007) cryopreserved common carp sperm in 5 mL straws using a Styrofoam box and a sugar-based extender. The results showed a higher hatching rate (65 ± 18%) than in our study (42%) when 1 straw was used for 40 g of eggs. An identical high hatching success (60 ± 2%) was recorded using 1.2 mL straws for fertilization at a laboratory scale (300 ± 10 eggs) by Lahnsteiner et al. (2003). Our results indicate that not enough viable and moving cells were available following the thawing process for effective fertilization of 1 kg of eggs. Former studies in common carp highlighted that sperm motility and swimming velocity have main effect on the variability of fertilization and hatching rate (Fauvel et al. 2010; Kaspar et al. 2008). Despite to the reduced motility parameters obtained following thawing, a moderate hatching rate was observed in our study. The phenomenon was caused by the sufficient amount of spermatozoa provided by the propagation protocol used in our experiment.

In our study, a relatively high percentage of malformed larvae were recorded. In contrast, a low severity and variety of the occurring malformations was observed possibly caused by reduced number of collected individuals. The most typical anatomical abnormalities were deformed tail development and head deformity. However, this phenomenon is not thought to be caused solely by the cryopreservation process, as malformation occurred at a similar proportion in the control group. In general, skeletal morphological changes occur both in wild populations and under farm conditions (Koumoundouros et al. 1997; Kužir et al. 2015; Slooff 1982). The prevalence of malformations in captive breeding is higher than in the natural environment (Cobcroft et al. 2001; Kužir et al. 2015). Former studies suggested that abnormalities are also induced during the embryonic and post-embryonic periods of life (Al-Harbi 2001). Various parameters can combine to result in skeletal and head deformities and injuries: genetics, cultivation conditions, nutritional deficiencies, environmental contamination, mechanical trauma, infectious, and parasitic diseases (Brown & Nuňez 1998; Eissa et al. 2009; Kužir et al. 2015; Milton 1971; Quigley 1995; Vogel 2000). In our study, the genetic background of the fish, as well as possible thermal shock (variations in the temperature and quality of the input water in the flow-through system) could have had a great effect on the malformation rate of hatched larvae. Further studies on selective breeding and embryo incubation (using different water temperatures and quality) may support our hypothesis (Brown & Nuňez 1998; Eissa et al. 2009; Kocour et al. 2006; Milton 1971; Vogel 2000). Interestingly, a similar dominance of head deformities (craniofacial and eye) was detected in ide (*Leuciscus idus*) larvae originating from fertilization using cryopreserved sperm (Bernáth et al. 2018; Cabrita et al. 2014). In present study, deformed tail development was also relevant in the cryopreserved group. Cryopreservation can affect the chromatin stability of fish sperm and DNA integrity has a high impact on early embryo development (Bernáth et al. 2018; Cabrita et al. 2014). Further studies are required to analyse the specific gene regions injured during the cryopreservation process that cause the specific head malformation in common carp and other cyprinids (Bernáth et al. 2018; Cabrita et al. 2014).

To our knowledge, this is the first report on the growth rate of common carp larvae (limited information available in other species as well) originating from frozen sperm reared in traditional pond culture. Growth parameters of larvae reported in former studies were obtained using fresh sperm for fertilization. Cryopreservation did not have a significantly negative effect on the growth rate of common carp larvae either in the pre-nursing or grow-out periods. A rapid increase in average body weight and total length was recorded during our experiment. This growth tendency was in accordance with the observations presented by Horváth et al. (2015). In their previous studies, these authors confirmed that in traditional pond culture, the live body weight and total length vary between 0.2–0.3 g and 2.5–3 cm, respectively, at the end of the rearing period (Horváth et al. 2002, 2015). A similar average total length (2.5–3 cm) was presented by Billard et al. (1995) for 1-month-old common carp larvae. The final weight of common carp fry described by the FAO (2009) is similarly between 0.2 and 0.5 g at the end of the nursing period. In our experiment, identical body parameters of common carp fry were observed in both controls (body weight: 0.5 ± 0.2 g, total length: 3 ± 0.3 cm) and cryopreserved (body weight: 0.5 ± 0.3 g, total length: 3 ± 0.4 cm) groups at the end of the pre-nursery period. Similarly, the growth rate was not affected by our cryopreservation method during the grow-out period. The body weight (fresh: 64 ± 19 g, cryopreserved: 66 ± 30 g) and total length (fresh: 16 ± 2 cm, cryopreserved: 16 ± 2 cm) measured at the end of the grow-out period were in accordance with the values (body weight: 10–40 g, total length: 8–12 cm) presented in the former study by Horváth et al. (2015). The successful fingerling production in both groups was supported by the data presented in FAO 2009 (common carp fingerling body weight: 30–100 g). In contrast, a lower final weight (22 g) of common carp fingerlings (using fresh sperm for propagation) was reported in Uzbekistan (Akromov et al. 2022). According to our study, common carp larvae originating from propagation using cryopreserved sperm showed a similar growth rate in traditional pond culture as the larvae originating from propagation using fresh sperm. Further studies should investigate the growth tendencies of common carp fingerlings (from frozen sperm) after the wintering period until they reach market size.

A significantly reduced survival rate of pre-nursed fish was detected in the cryopreserved sperm group in comparison to the fresh sperm control group. This period lasts on average 21–30 days, with the survival of individuals dependant on several factors: (1) water temperature, (2) quantity and quality of natural foods (and supplementary feed), (3) oxygen, (4) predators, and (5) water conditions (Horváth et al. 2015). According to the observation of the hatchery director at the fish farm, the pond selection and preparation had a major effect on the fish survival rate at the end of the pre-nursing period (more predator organisms) in the frozen sperm group. The observation (pond selection and preparation has an effect on fish survival rate) was also proved in the former studies by Horváth et al. (2002 and 2015). Zooplankton is the most important feed source at the beginning of the pre-nursing period. However, inadequate pond preparation can limit the availability of essential organisms (e.g. rotifers) and thus allows for the growth of dangerous crustaceans (e.g. copepods) (Horváth et a. 2015). Nevertheless, the survival rate in both groups (fresh: 72%, cryopreserved: 43%) was in accordance with the expected average result (30–70%) of larvae pre-nursery survival rates in traditional pond cultures (Horváth et al. 2002 and 2015). Interestingly, this difference was not detectable during the grow-out period in the fresh and frozen groups. The less sensitive, advanced fry showed a higher survival rate (fresh: 95%, cryopreserved: 96%) compared to the data available in former studies (25–80%; Horváth et al. 2002 and 2015).

## Conclusions

Large-scale cryopreserved sperms were effectively applied in the fertilization of large (industrial-scale) amounts of common carp eggs. According to our findings, the handling of large numbers of high-volume straws during the thawing process is difficult and can notably affect the fertilization rate. Future innovations (technical and technological also) will hopefully allow for the establishment of standardized methods (or equipment) to provide a more controlled environment for thawing process. Novel tendencies were observed in hatching and malformation rate regardless of the lowered sample size. Despite the limited available rearing ponds (1–1) provided by the commercial company, the equal growth and viability in larvae obtained from fertilization using cryopreserved sperm were recorded for the first time in traditional pond culture. Importantly, proper pond selection and preparation should be considered as a limiting factor to successful fry production during the pre-nursing period. Furthermore, future experiments could reveal the detailed effects of the hatchery progress and environment on the dynamics of larvae (obtained from cryopreserved sperm) growth in an elevated number of traditional rearing ponds.

## Data Availability

The dataset presented in this study is available from the corresponding author following direct request.
